# Gestational stress decreases postpartum mitochondrial respiration in the prefrontal cortex of female rats

**DOI:** 10.1016/j.ynstr.2023.100563

**Published:** 2023-08-14

**Authors:** Erin Gorman-Sandler, Breanna Robertson, Jesseca Crawford, Gabrielle Wood, Archana Ramesh, Olufunke O. Arishe, R. Clinton Webb, Fiona Hollis

**Affiliations:** a Department of Pharmacology, Physiology, and Neuroscience, University of South Carolina School of Medicine, Columbia, SC, USA; b Columbia VA Health Care Systems, Columbia, SC, 29208, USA; c Department of Cell Biology and Anatomy, University of South Carolina School of Medicine, Columbia, SC, USA; d Cardiovascular Translational Research Center, University of South Carolina School of Medicine, Columbia, SC, USA; e USC Institute for Cardiovascular Disease Research, Columbia, SC, USA

**Keywords:** Chronic unpredictable stress, Prefrontal cortex, Pregnancy, Postpartum, Susceptibility, Mitochondria

## Abstract

Postpartum depression (PPD) is a major psychiatric complication of childbirth, affecting up to 20% of mothers, yet remains understudied. Mitochondria, dynamic organelles crucial for cell homeostasis and energy production, share links with many of the proposed mechanisms underlying PPD pathology. Brain mitochondrial function is affected by stress, a major risk factor for development of PPD, and is linked to anxiety-like and social behaviors. Considering the importance of mitochondria in regulating brain function and behavior, we hypothesized that mitochondrial dysfunction is associated with behavioral alterations in a chronic stress-induced rat model of PPD. Using a validated and translationally relevant chronic mild unpredictable stress paradigm during late gestation, we induced PPD-relevant behaviors in adult postpartum Wistar rats. In the mid-postpartum, we measured mitochondrial function in the prefrontal cortex (PFC) and nucleus accumbens (NAc) using high-resolution respirometry. We then measured protein expression of mitochondrial complex proteins and 4-hydroxynonenal (a marker of oxidative stress), and Th1/Th2 cytokine levels in PFC and plasma. We report novel findings that gestational stress decreased mitochondrial function in the PFC, but not the NAc of postpartum dams. However, in groups controlling for the effects of either stress or parity alone, no differences in mitochondrial respiration measured in either brain regions were observed compared to nulliparous controls. This decrease in PFC mitochondrial function in stressed dams was accompanied by negative behavioral consequences in the postpartum, complex-I specific deficits in protein expression, and increased Tumor Necrosis Factor alpha cytokine levels in plasma and PFC. Overall, we report an association between PFC mitochondrial respiration, PPD-relevant behaviors, and inflammation following gestational stress, highlighting a potential role for mitochondrial function in postpartum health.

## Graphical abstract

**Figure:**
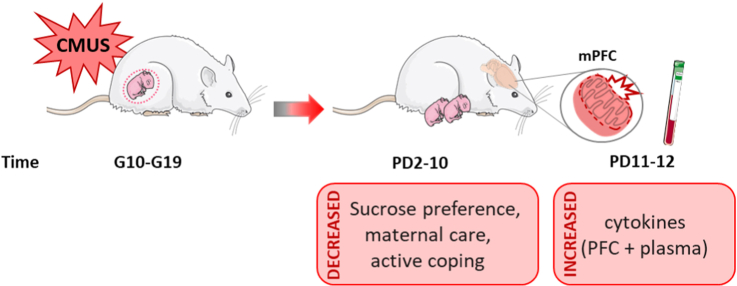

## 1 Introduction

Pregnancy and the postpartum period are accompanied by a high risk for complications which may negatively affect both mother and child. Mood alterations are a common complication, with depressive symptoms reported in 70% of women during pregnancy (Becker et al., 2016; O'Hara and Wisner, 2014). Furthermore, up to 1 in 5 women experience severe depressive symptoms within the year following parturition, often resulting in the diagnosis of postpartum depression (PPD) (“Depression Among Women | Depression | Reproductive Health | CDC,” 2020; O’Hara and Swain, 1996). While PPD overlaps major depressive symptoms such as anhedonia, loss of energy, sleep disturbances, social dysfunction, cognitive impairment, feelings of hopelessness, and comorbid anxiety, it is further characterized by hallmark disturbances in the mother-infant relationship which include a lack of interest in the child, difficulty bonding, and in severe cases, thoughts of harm towards the self or child (O'Hara, 2009). As such, PPD accounts for the majority of postpartum deaths in the form of suicide (Becker et al., 2016) and is associated with impairments in maternal care which can negatively affect child development and increase their risk for psychiatric disturbances later in life (O'Hara, 2009). Although PPD contributes to increased health risks in both mother and child, with potentially long-term consequences, the underlying neurobiology of PPD remains unclear and effective treatment options are limited.

Stress is one of the largest risk factors for developing PPD, outside of a previous history of depression (Qiu et al., 2020), with negative, stressful life events associated with increased depressive symptoms (Lancaster et al., 2010). Moreso, women with PPD may have altered hypothalamic-pituitary-adrenal (HPA) axis function (Jolley et al., 2007). As such, animal models based on gestational chronic stress paradigms or corticosterone (CORT) injections administered during gestation or postpartum period induce PPD-relevant behaviors, such as altered maternal care and anhedonia (Brummelte and Galea, 2010; Leuner et al., 2014; O'Mahony et al., 2006; Pardon et al., 2000; Perani and Slattery, 2014). These behavioral outputs have been associated with molecular changes in inflammatory responses, hormone signaling, neuroplasticity, and neurotransmission (Brummelte and Galea, 2016; Corwin et al., 2015; McEwen et al., 2012; Pawluski et al., 2011, 2016; Schiller et al., 2015). For example, stress-sensitive regions such as the prefrontal cortex (PFC), nucleus accumbens (NAc), and hippocampus (HPC) demonstrate structural changes and increased cytokine levels in animal models of postpartum depression (Dye et al., 2022; Haim et al., 2014; Leuner et al., 2014; Workman et al., 2013). Additionally, the steroid hormone progesterone and its metabolite and neurosteroid, allopregnanolone, are associated with adaptation to stress (Brunton et al., 2014). Both women with PPD (Walton and Maguire, 2019) and rodents exposed to chronic stress (Bali and Jaggi, 2014) exhibit decreased levels of allopregnanolone. Recent work found that treatment with a synthetic formulation of allopregnanolone that targets GABA_A_ receptors, Brexanolone, was highly successful in reversing PPD symptoms in humans (Kanes et al., 2017), though the long-term mechanism is unclear (Walton and Maguire, 2019) and the current treatment regimen is expensive and intensive. GABA hypofunction has been observed in both humans and rodent models of PPD (Maguire and Mody, 2008; Walton and Maguire, 2019) and treatment with Brexanolone acutely reverses this deficit in rodents (Melón et al., 2018). Interestingly, these molecular processes are all intimately linked to mitochondrial function (Accardi et al., 2014; Bordt et al., 2019; Bordt and Polster, 2014; Cheng et al., 2010; Du et al., 2009; West, 2017), suggesting a potential role for mitochondria in the stress-induced alterations observed in PPD.

Mitochondria are essential organelles that produce energy to facilitate cellular, physiological, and behavioral responses (Picard et al., 2018). The brain consumes approximately 20% of the body's total energy (Rettberg et al., 2014), and stress can amplify that need, leading to increased mitochondrial activity to meet energy demands (Morava and Kozicz, 2013; Picard et al., 2018). Thus, mitochondria have been recently identified as vital for stress adaptation and may be involved in depressive pathology, with even small deficits in function producing impairments in synaptic plasticity and neurogenesis (Brinton, 2008; Morava and Kozicz, 2013; Picard et al., 2018; Rettberg et al., 2014). Preclinical studies in male rodents found that chronic stress reduced mitochondrial function in the cortex and cerebellum (Madrigal et al., 2001; Rezin et al., 2008). Importantly, however, the effects of gestational stress and parity on brain mitochondria in postpartum females remains completely unexplored. We previously demonstrated a direct role for mitochondrial function within the NAc and PFC in anxiety-like and social behaviors (Hollis et al., 2015, 2018) revealing an active role for these organelles in regulating behavioral phenotypes relevant to depression. Moreover, mitochondria release immunogenic compounds that stimulate the release of pro-inflammatory cytokines (West and Shadel, 2017) that have been implicated in PPD (Dye et al., 2022). Combined with the involvement of mitochondria in many of the physiological changes that occur during pregnancy and parturition, as well as their vital role in stress and inflammation, mitochondria have great potential to mediate postpartum-induced alterations in brain and behavior, and may contribute to susceptibility in the development of PPD.

We sought to determine whether gestational stress exposure impacts mitochondrial function in specific brain regions implicated in PPD and mitochondrial mediation of behavior. We exposed pregnant female rats to chronic mild unpredictable stress (CMUS) during late gestation, and assessed postpartum behaviors relevant to PPD, brain mitochondrial respiration, and cytokine levels. We hypothesized that CMUS will induce postpartum depressive-like behaviors and decrease brain mitochondrial function within stress-sensitive regions implicated in PPD, specifically the PFC and NAc that may associate with increased cytokine levels. We found that gestational CMUS altered postpartum behaviors, and interestingly, decreased mitochondrial respiration in the PFC but not in the NAc. These findings provide rationale to further investigate the role of brain mitochondria in stress-induced pathologies.

## 2 Methods

### 2.1 Animals

**Fig. 1:**
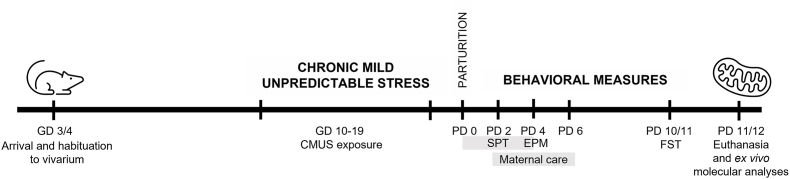
**Experimental Timeline.** Nulliparous and primiparous female rats were weighed and handled upon arrival at the vivarium. Females were weight-matched (within parity groups) and assigned to stress or non-stress conditions. Chronic mild unpredictable stress (CMUS) began on the day corresponding to gestational day (GD) 10 in primiparous rats and continued for 10 days through GD 19. Following parturition on GD 21/postpartum day (PD) 0, a series of behavioral tests were performed during the postpartum period. The sucrose preference test (SPT) was performed from PD 0–4, the elevated plus maze (EPM) was performed on PD 4, maternal care was assessed during the light cycle and at the onset of the dark cycle on PD 2, 3, 5, and 6, and the one-session forced swim test (FST) was performed on PD 10 or 11. All rats that underwent behavior were euthanized on the days corresponding to PD 11 or 12 and mitochondrial respiration was measured in the prefrontal cortex and nucleus accumbens. Other molecular analyses (protein and cytokine measurements) are representative of this same euthanasia timepoint.

Intact and cycling nulliparous and timed mated primiparous adult female Wistar rats (Envigo, Virginia, USA) initially weighing 225–250g arrived at the vivarium in the same delivery day and were used in these experiments. Thus, experimental time points for both nulliparous and timed-mated females are matched to the primiparous female gestational day (GD). Upon arrival on gestational day 4 (GD4), rats were housed individually in standard polycarbonate rat cages on a 12:12h light/dark cycle (lights on at 07:00h) and provided food and water ad libitum. Single-housing allowed for individual home cage assessments of behavior (such as the sucrose preference test and maternal care). All animals were weighed on arrival and handled for at least three days before the start of any manipulations. Stress manipulations began 1 week after arrival to allow for habituation to the vivarium (see Fig. 1 for an experimental timeline).

Animals were weight-matched and assigned to the following experimental groups:

1) Nulliparous/non-stressed (control: C), n = 11
2) Nulliparous/stressed (stress only: S), n = 11
3) Primiparous/non-stressed (primiparous only: P), n = 9–13
4) Primiparous + stressed (P + S), n = 9–12

The inclusion of C, S, and P groups allow us to account for potential individual effects of stress and pregnancy on weight, behavior, and mitochondrial respiration. Groups exposed to stress were housed in a separate room from non-stressed groups. After the start of stress, all animals were weighed every other day and cage changes were performed twice weekly, except on the days surrounding behavioral testing. During behavioral testing, cage changes were avoided the day before a behavioral measure to limit potential effects on behavior. All behavioral measurements were performed between 08:00 and 14:00h, with the exception of the home cage sucrose preference test and maternal care observations (see description below under “Sucrose Preference Test” and “Maternal Care”). The day of parturition was designated as postpartum day 0 (PD 0). Litters were characterized briefly for number of pups and sex ratio (Table 1). All efforts were made to minimize animal suffering and reduce the number of animals used where possible. All experimental procedures were approved by The University of South Carolina Institutional Animal Care and Use Committee and conformed to the U.S. National Institutes Health Guide for the Care and Use of Laboratory Animals.

**Table 1:** **Litter characteristics.** Primiparous dams had no significant differences in litter size, litter weight, sex ratio, or mortality rate (*Unpaired two-tailed student's t-test*; with *Welch's correction* applied for analysis of pup mortality).

**Measure**	**P (n=9)**	**Std dev.**	**P + S (n=9)**	**Std dev.**	**p-value**	**t-value**	**df**
**Avg litter size (#)**	11.2	4.969	11.7	3.606	0.8308	0.2172	16
**Avg litter weight (g)**	8.6	0.805	8.4	0.6815	0.5437	0.618	20
**Avg percentage of male offspring (%)**	46.5	11.72	55.03	16.6	0.2359	1.235	15
**Pup mortality (#)**	0	0	0.1111	0.3333	0.3466	1	8

### 2.2 Chronic mild unpredictable stress (CMUS)

Nulliparous (S) and primiparous (P + S) groups were exposed to a chronic, mild, unpredictable stress (CMUS) protocol, adapted from Frisbee et al. This protocol has emerged as a highly translationally-relevant model for studying the pathophysiology of chronic stress exposure in rodents (Willner, 2017; Willner et al., 1987) A fundamental concept of the CMUS protocol that enhances its relevance to human situations lies in the heterotypic, unpredictable and uncontrollable nature of the stressors that prevents adaptation and leads to disrupted stress response systems (Frisbee et al., 2015). Our protocol consisted of a randomized series of daily exposures to one of the following stressors (Table 2): white noise, bedding alterations (wet bedding, no bedding), tilted cage, overnight light exposure, and acute restraint stress during the second half of gestation (GD 10–19), to avoid altered reproductive effects (Gemmel et al., 2018). The length of exposure for each stressor, as well as the time of day that each was introduced was varied to maximize unpredictability and uncontrollability. Both S and P + S groups began stress exposure on the same day and experienced the same order and timing of stressors. The last stress manipulation occurred on GD 19. PPD-relevant behaviors were measured following parturition, after cessation of stress.

**Table 2:** **Chronic Mild Unpredictable Stress paradigm.** Nulliparous and primiparous female rats in the stress group began stress exposure on the day corresponding to GD 10. Stress exposure occurred over varying lengths of time and at different times of day, and included wet bedding, no bedding, overnight light, white noise, cage tilt, and restraint stress, as detailed in table. All cohorts followed the same stress schedule.

Day	Stressor	Duration
GD10	Wet bedding	3h
GD11	Tilted cage (30° angle)	5h
GD12	Overnight light	12h
GD13	Restraint stress	3h
GD14	Bedding removed	4h
GD15	Wet bedding	7h
GD16	White noise	6h
GD17	Overnight light	12h
GD18	Restraint stress	1h
GD19	Wet bedding	4h

### 2.3 Behavioral testing

#### 2.3.1 Sucrose preference test

The sucrose preference test, a two-bottle choice paradigm, was performed according to the procedure outlined by Bolaños et al. (2008) and our lab (Hollis et al., 2010, 2011). This test has been used extensively in evaluating stress-induced anhedonia (Willner, 2017; Willner et al., 1987), a core symptom of depression characterized by lack of ability to experience pleasure. Rats were habituated to drink water from two 250 mL bottles for four days. A water intake baseline was measured for 48 h within the week prior to the start of sucrose introduction in order to assess differences in fluid intake. From PD0-4, animals were exposed to ascending concentrations of sucrose (0.25% and 1%) for two days per concentration. Animals were group matched, and the exact timing of when the test began was dependent on time of parturition. Notably, all pregnant dams gave birth within 24 h of each other. The amount of water and sucrose solution consumed was measured daily by weighing bottles. The positions of the bottles in each cage were switched between the groups 1–3 times daily to eliminate effects of side preference. The preference for sucrose over water was used as a measure of the rats’ response to a naturally rewarding stimulus. Specifically, sucrose preference was measured as (sucrose intake (grams)/total water + sucrose intake (grams)) x 100%. Preference significantly lower than controls was considered anhedonic behavior.

#### 2.3.2 Elevated plus maze

On PD 4, all groups were tested for anxiety-like behavior in the elevated plus maze. Rats were habituated to the testing room for at least 30 min. The apparatus was elevated (50 cm) from the floor and consisted of two closed arms (50 x 10 × 40 cm), two open arms (50 × 10 cm), and was separated by a center zone (10 × 10 cm). Lighting was maintained at 4–7 lux in the closed arms, 18–21 lux on the open, and 12–15 lux in the center. These lighting conditions ensure observations of trait anxiety as opposed to state anxiety, as very bright lighting can itself be anxiogenic (Hollis et al., 2015). Animals were placed in the center of the maze facing a closed arm, and behavior on the maze was recorded for 300s. Before testing and in between trials, the apparatus was cleaned with a 10% EtOH solution. Distance moved, average velocity, and time spent in the center, open arms, and closed arms were measured. Anxiety-like behavior was determined by % time spent in the open arms. (Time spent in open arms (sec)/total time of test(sec)) x 100%. Behaviors were scored using automated scoring by EthoVision XT.

#### 2.3.3 Maternal care: home cage nesting observations

Home cage nesting observations were used to assess maternal behaviors in basal conditions from PD2-6, as alterations in maternal care are characteristic of a gestationally stressed phenotype. PD4 recording was excluded as behavioral testing interfered with recordings. Dams were recorded in the light cycle (12:00-13:00h), and the dark cycle (19:00-20:00h) for 1 h each using a video recorder. Red lighting was used to allow for scoring during dark cycle recordings. Cages of P and P + S dams were turned with the nest facing the video recorder. The experimenter was not present during the recording period, and videos were scored at a later time. Scored behaviors included the number of licking and grooming, nursing, eating and drinking, and self-grooming events. Scoring was performed manually by a trained experimenter blind to the experimental conditions using Noldus Observer XT15. Videos were instantaneously sampled every third minute across each 60-min session and 20 total events per hour per day were recorded and analyzed, resulting in a total of 160 observations per rat. We report the behaviors averaged across postpartum days and times.

#### 2.3.4 Forced swim test

The forced swim test was performed on the day preceding euthanasia (PD10 or 11) to characterize passive versus active coping style. Active coping is associated with more time spent swimming/struggling, while passive is associated with more time spent immobile (Molendijk and de Kloet, 2015). Animals were brought to the behavioral room to habituate for at least 30 min. Cylindrical tanks (60 cm in height) were filled with fresh water, at a temperature between 24 and 26 °C. Animals were individually placed into the tank and underwent swim exposure for a total of 15 min. After the 15-min test, animals were taken out of the tank and dried by towel and space heater before being returned to their individual home cages. The entire procedure was video-recorded, and behavior was later quantified manually by an experimenter blind to the treatments, for latency to become immobile and time spent immobile. Latency to immobility was defined as the time at which the rat first adopted a stationary pose not associated with an attempt to escape. Immobility was manually scored by a trained individual blind to treatment groups using Noldus Observer XT15. Immobility was defined as when the rat remained in this stationary posture for more than 2.0 s, making only the movements necessary to maintain its head above water.

### 2.4 Euthanasia and tissue collection

Animals were euthanized on PD 11 or 12 under basal conditions by rapid decapitation. Importantly, this mid-postpartum time point corresponds with a return of estradiol levels to basal levels, following a peak at the end of gestation that steadily decreases through PD 14 (Camacho-Arroyo et al., 2018). Trunk blood was collected in EDTA-coated tubes (Sarstedt) and immediately chilled in ice. Plasma was isolated from blood via centrifugation at 16,000×*g* for 3 min. Plasma was immediately snap frozen in chilled isopentane and stored at −80 °C until further processing. The brain was rapidly removed and PFC and NAc dissected out. One hemisphere from each region was snap frozen in chilled isopentane and stored at −80 °C. The remaining hemisphere was weighed and placed in a well plate on ice with 2 mL of relaxing solution (BIOPS: 2.8 mM Ca2K2EGTA, 7.2 MK2EGTA, 5.8 mM ATP, 6.6 mM MgCl2, 20 mM taurine, 15 mM sodium phosphocreatine, 20 mM imidazole, 0.5 mM dithiothreitol and 50 mM MES, pH = 7.1) until further processing.

### 2.5 Mitochondrial respirometry

Tissue samples were processed as described in (Hollis et al., 2015). Briefly, samples were gently homogenized in ice cold MiR05 respirometry medium (0.5 mM EGTA, 3 mM MgCl2, 60 mM potassium lactobionate, 20 mM taurine, 10 mM KH2PO4, 20 mM HEPES, 110 mM sucrose and 0.1% (w/v) BSA, pH = 7.1) with a motorized Teflon pestle. One hemisphere of each isolated region of isolated PFC (approximately 20–30 mg) or NAc (approximately 20 mg) tissue was homogenized and subsequent PFC or NAc tissue homogenates were used to measure mitochondrial respiration rates at 37 °C using high resolution respirometry (Oroboros Oxygraph 2K, Oroboros Instruments, Innsbruck, Austria). A multi-substrate protocol was used to sequentially explore the various components of mitochondrial respiratory capacity as previously described (Hollis et al., 2015). Briefly, samples were normalized to wet weight and 2 mg of tissue homogenate were loaded into a 2 mL chamber at 37C. Coupled respiration through complex I was stimulated by the addition of ADP (5 mM) to a mixture of malate (2 mM), pyruvate (10 mM) and glutamate (20 mM). Coupled respiration through complexes I and II (CI + CII) was stimulated by the addition of succinate (10 mM). Uncoupled respiration to examine the maximal capacity of the electron transport system (ETS) was measured using the protonophore, carbonylcyanide m-chlorophenyl hydrazone (CCCP; successive titrations of 0.2 μM until maximal respiration rates were reached). We then examined consumption in the uncoupled state due solely to the activity of complex II by inhibiting complex I with the addition of rotenone (0.1 μM; ETS CII). Finally, electron transport through complex III was inhibited by adding antimycin (2 μM) to obtain the level of residual oxygen consumption (ROX) due to oxidating side reactions outside of mitochondrial respiration. The O_2_ flux obtained in each step of the protocol was corrected for residual oxygen consumption (ROX). In line with other published reports (Burtscher et al., 2015), we use CI + II/ETS-linked capacity as an internal standardization factor to control for differences in mitochondrial density between samples. CI + II/ETS-linked capacity encompasses many influential factors of respirometry and significantly correlates with citrate synthase activity, a common measure of mitochondrial density (Burtscher et al., 2015). Unlike citrate synthase which typically is performed on a separate sample in an independent assay, CI + II/ETS-linked capacity is calculated from the same sample and thus potentially a better representation of mitochondrial density within that specific sample.

### 2.6 Protein expression

**Supplementary Fig. 1:**
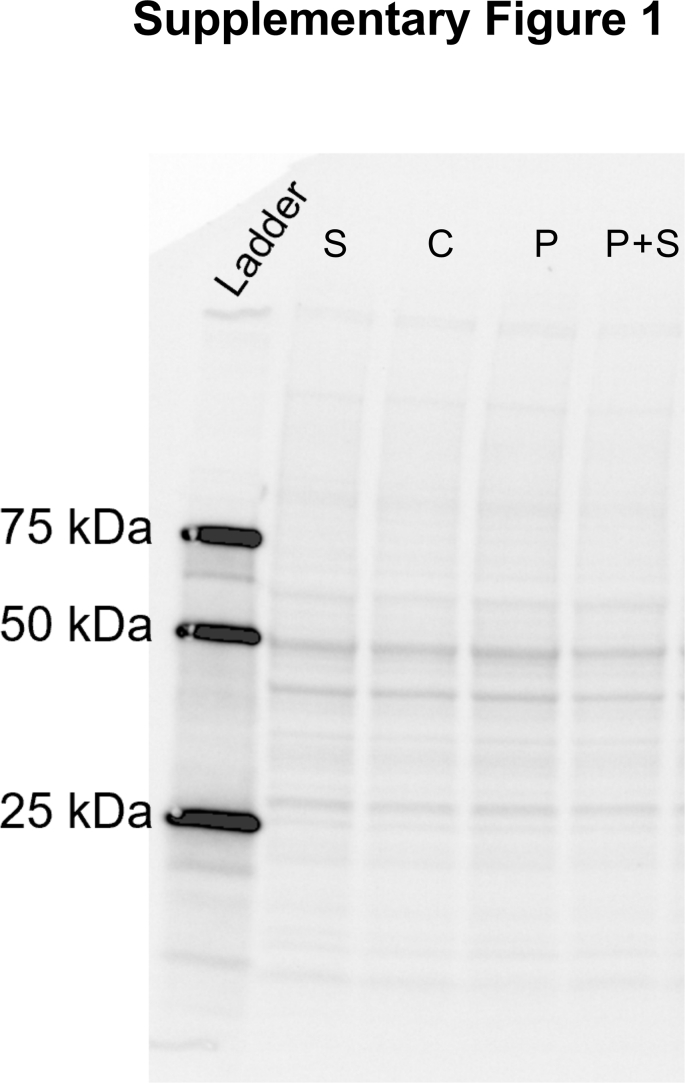
**Representative total protein image of stain-free western blot (post-transfer)**. C = nulliparous controls, S = nulliparous stressed, P = primiparous controls, and P + S = primiparous stressed.

Relative protein expression was assessed using Western blotting technique, with primary antibodies (AB) against individual subunits of each complex in the oxidative phosphorylation (OXPHOS) system, 4-hydroxynonenal (4-HNE), a marker of oxidative stress, and Translocase of the Outer Membrane 20 (TOM20), a receptor protein located on the outer mitochondrial membrane that is a component of the import receptor complex that translocates cytoplasmically synthesized mitochondrial proteins into mitochondria (Yamamoto et al., 2011). TOM20 is a commonly used marker for mitochondrial content (Zhang et al., 2013). Protein concentration of PFC homogenates in respirometry medium was determined by BCA assay (ThermoScientific, Cat. #23227). Samples were prepared with Laemelli buffer and milliQ water to a final concentration of 1 μg/μL, then heated in a dry bath for 5 min at either 37 °C (for OXPHOS AB detection) or 95 °C (for 4-HNE and TOM20 AB detection). 20 μL of protein lysate was then loaded into 4–20% Criterion™ TGX Stain-Free™ precast gels (Bio-Rad, Cat. #5678094). Proteins were separated at 200V for 45 min and then transferred to a low-fluorescence PVDF membrane at 25V for 30 min using the Trans-Blot Turbo Transfer System and Kit (Bio-Rad, Cat. #1704275). Gels were activated by UV excitation using the Bio-Rad GelDoc XR + System prior to transfer, and membranes were imaged for total protein on the same system following transfer according to manufacturer's instructions. The use of TGX Stain-Free gels allows for visualization and quantification of total protein across all lanes similar to that provided by Coomassie staining. Total protein was determined by quantifying the two most distinct bands (at 37 kDa and 50 kDa) and summing the values, as they are representative of the total staining and consistent across samples (see representative stain in Supplementary Fig. 1. Membranes were then blocked with EveryBlot Blocking Buffer (Bio-Rad Cat. #12010020) for 30 min (for 4-HNE and TOM20 AB detection) or 1 h (for OXPHOS AB detection) and then incubated overnight at 4 °C with Rodent Total OXPHOS antibody cocktail (Mitosciences, ab110413; 1:250), anti-4-Hydroxynonenal (Sigma-Aldrich, AB5605; 1:4000), or TOM20 (Abclonal, A19403; 1:2000) in blocking buffer. Following washes in PBS-T (VWR, Cat. #76371-736), membranes were incubated with IRDye® 680RD Goat-anti-Mouse (LI-COR, #926–68070) or IRDye® 800CW Donkey anti-Goat (LI-COR, #926–32214), or IRDye® 800 CW Goat anti-Rabbit (LI-COR, #926–32211) IgG secondary antibodies at a 1:20000 dilution for 1h at room temperature. After PBS-T washes, bands were captured using a LI-COR Odyssey CLX Imager. Bands were quantified as 16-bit TIF images using ImageJ software (NIH). The 4-HNE antibody binds to HNE-modified protein adducts resulting from lipid peroxidation. Thus, this antibody results in multiple bands within each lane, which were all quantified. Quantified bands were normalized to total protein to control for variance in sample loading. Western blot data are presented as fold change relative to control averages to control for between-blot variability.

### 2.7 Assessment of central and peripheral cytokine levels

Cytokine levels were assessed using Bio-Rad BioPlex assays in both plasma and PFC homogenates (Th1/Th2 rat cytokines Bio-Rad, #171k1002M), according to manufacturer's protocols. PFC homogenates were diluted 1:3 and plasma were diluted 1:4 with diluent. Plates were read on a Luminex plate reader using high photomultiplier voltage and analyzed with Bio-Plex manager software. PFC cytokine levels were normalized to tissue protein.

### 2.8 Statistical analyses

Sample sizes are indicated in the figure legends. Experiments were run in 3 independent cohorts of n = 3–5/group. While all cohorts followed the same experimental timeline, technical issues resulted in variations in samples sizes across measures. For example, maternal care data are presented from cohorts 2 and 3 only as cohort 1 recordings were too dark to identify specific maternal behaviors. Additionally, sample sizes within behavioral and respirometry measures vary due to missing data for behavior-specific reasons (e.g., 1 animal was excluded from sucrose preference as the bottle leaked overnight and a preference could not be determined) or as statistically significant outliers (determined from the Grubbs Outlier Test – 2 rats excluded from mitochondrial respirometry, and 1 rat excluded from cytokine assays).

Data were analyzed by unpaired Student's t-test (maternal care and litter parameters), one sample *t*-test (sucrose preference test against indifference point) or two-way ANOVAs (all other behaviors, protein expression data, and cytokine data), followed by *Šídák's* multiple comparisons post hoc test where appropriate. Two-way ANOVAs tested stress exposure and pregnancy as between-subject factors. Correlational data were analyzed using a simple linear regression. Figures are presented with original, non-transformed data. All data were analyzed using Prism version 9 (GraphPad software Inc., San Diego, CA). P-values are reported in figure legends and related statistical details are collated into Supplementary Tables 1 and 2 Statistical significance was considered at the p < 0.05 level.

## 3 Results

### 3.1 Validation of gestational CMUS paradigm

We first validated that our CMUS protocol successfully altered postpartum behaviors relevant to PPD. In rodent studies, several groups report that exposure to gestational stress induces reduced gestational weight gain, anhedonia, reduced or disrupted maternal care, and passive coping in the forced swim test (Haim et al., 2014; O'Mahony et al., 2006; Pardon et al., 2000; Smith et al., 2004; Zhang et al., 2022; Zoubovsky et al., 2020). Some report evidence of increased anxiety-like behavior (Darnaudéry et al., 2004; Hillerer et al., 2011), while others observe no effect (Leuner et al., 2014). Thus, we selected these same behaviors for assessment in our animals to ensure that our application of the CMUS protocol was effective. Note that statistical details including type of statistical test, p-values, as well as F- and t-statistics are reported for all main figures (Supplementary Table 1) and supplementary figures (Supplementary Table 2).

**Fig. 2:**
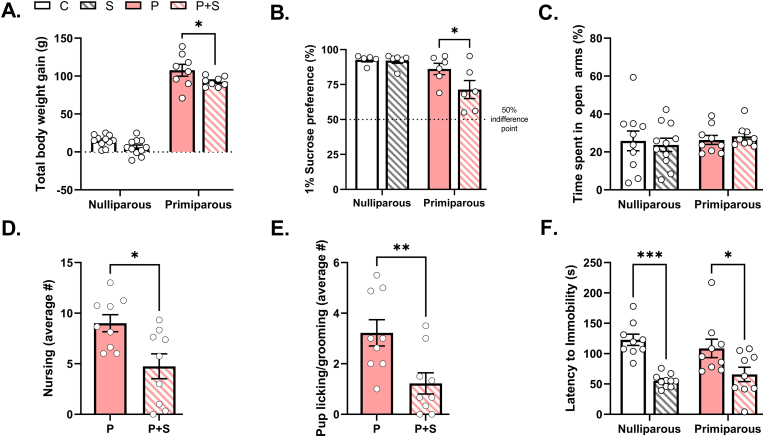
**CMUS successfully induced PPD-relevant behaviors in gestationally stressed dams. (A)** P + S dams had significantly lower body weight gain during GD 10–19 (stress period) than P dams (p = 0.04), while S females did not significantly differ in body weight gain from C females, as evidenced by significant main effects of both stress (p = 0.006) and parity (p < 0.0001), but no significant interaction between stress and parity (p = 0.4). n = 8–11/group. **(B)** There was a significant main effect of parity on sucrose preference (p = 0.004), and trends for a main effect of stress (p = 0.08), where P + S dams exhibited significantly lower preference for a 1% sucrose solution compared to primiparous controls (p = 0.04). There was no statistically significant interaction between stress and parity in 1% sucrose preference (p = 0.1). n = 5–6/group. **(C)** There was no significant interaction between stress and parity on percent time spent in open arms (p = 0.3), and no main effects of either parity (p = 0.5) or stress (0.98). n = 9–11/group. P + S dams had, on average, significantly reduced bouts of **(D)** nursing (p = 0.01) and **(E)** licking/grooming (p = 0.009) compared to P dams. n = 9/group. **(F)** There was a significant main effect of stress on both nulliparous and primiparous females in latency to immobility in the forced swim test (p < 0.0001), but no significant interaction (p = 0.3) or main effect of parity (p = 0.9). Both S and P + S groups had significantly shorter latencies to become immobile compared to respective controls (C vs. S: p = 0.0001, P vs. P + S: p = 0.02). n = 9–10/group. C = nulliparous controls, S = nulliparous stressed, P = primiparous controls, and P + S = primiparous stressed. All data are represented as mean ± SEM. *p ≤ 0.05, **p ≤ 0.01, ***p ≤ 0.001, ****p ≤ 0.0001.

We recorded weight gain across CMUS exposure, observing an expected significant main effect of parity on weight gain during the stress period, with primiparous groups gaining more total weight than nulliparous groups due to gestation (Fig. 2A). Within parity groups, primiparous + stress (P + S) dams had significantly reduced total body weight gain compared to their non-stressed primiparous (P) counterparts, while nulliparous control (C) and stress (S) groups did not differ from each other (Fig. 2A). Despite differences in weight gain, there were no differences in litter size, sex ratio, pup survival rate, or average pup weight (at postnatal day 4) between primiparous dams (Table 1).

**Supplementary Fig. 2:**
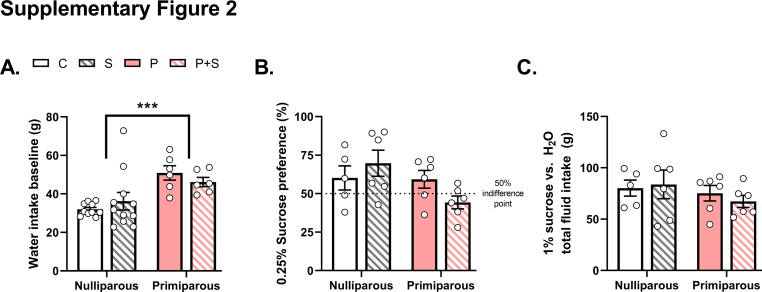
**Main effects of parity in water baseline and no effects of stress on 0.25% sucrose preference or total liquid consumption**. (A) Water intake baseline during the week prior to parturition exhibited a significant main effect of parity (p = 0.001) but no significant interaction (p = 0.23) or main effect of stress (p = 0.95) such that primiparous females had significantly higher water intake compared to nulliparous groups. n = 6–11/group. **(B)** Preference for a weak (0.25%) sucrose solution exhibited trends for a main interaction between stress and parity (p = 0.08) and main effect of parity (p = 0.06), but no significant main effect of stress (p = 0.69). n = 5–6/group. **(C)** Total fluid intake during 1% sucrose measurements were not significantly different between groups, evidenced by no main effects of parity (p = 0.27), stress (p = 0.82), nor interaction (p = 0.55). n = 5–6/group. C = nulliparous controls, S = nulliparous stressed, P = primiparous controls, and P + S = primiparous stressed. All data are represented as mean ± SEM. *p ≤ 0.05, **p ≤ 0.01,***p ≤ 0.001.

**Supplementary Fig. 3:**
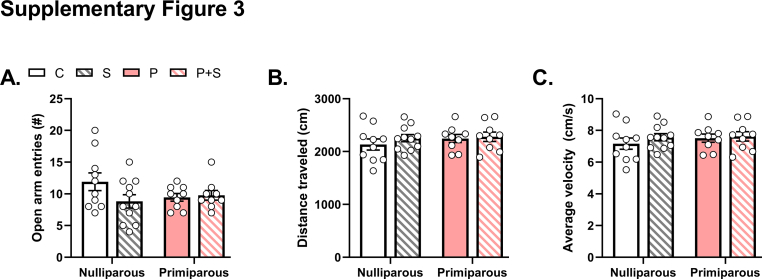
**No significant differences in open arm entries, distance, or velocity during the elevated plus maze**. (A) Open arm entries were not different between groups as evidenced by no main effects of parity, stress, nor interaction n = 9–11/group. **(B)** All groups traveled similar distances during testing, with no significant main effects of parity, stress, nor interaction. n = 9–11/group. **(C)** Average velocity was similar between groups. n = 9–11/group. See Supplementary Table 2 for statistical details. C = nulliparous controls, S = nulliparous stressed, P = primiparous controls, and P + S = primiparous stressed. All data are represented as mean ± SEM.

**Supplementary Fig. 4:**
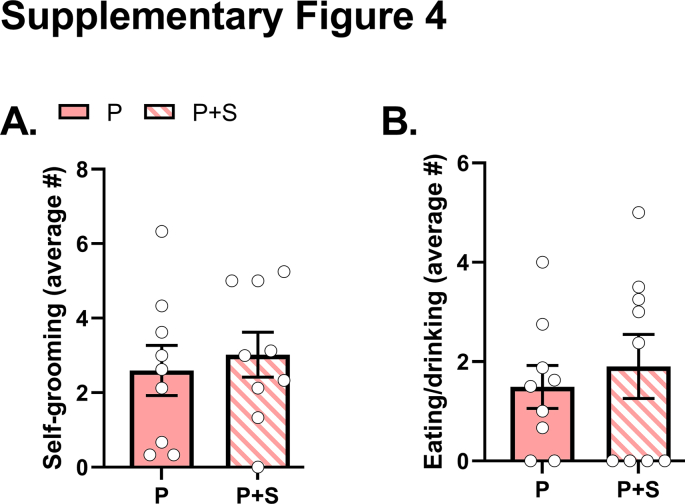
**No significant effects of stress on self-grooming or consummatory behaviors**. On average, P + S dams did not exhibit differences in **(A)** self-grooming (p = 0.65) **(B)** or consummatory (eating/drinking) behavior (p = 0.6) during home cage maternal care observations. n = 9/group. P = primiparous controls and P + S = primiparous stressed. All data are represented as mean ± SEM..

Following parturition, we assessed anhedonia in the early postpartum period using the sucrose preference test. Baseline measurement of water consumption revealed a significant effect of parity with both P and P + S females consuming more water than nulliparous groups (Supplementary Fig. 2A). In a first cohort of rats, assessment with 0.25% sucrose revealed trends for a significant effect of parity and interaction with stress (Supplementary Fig. 2B). However, preference was low across all groups and not significantly different from 50% (see Supplementary Table 1). When we measured preference for a higher concentration of sucrose solution (1%) in subsequent cohorts, we observed preferences significantly above the 50% indifference point (see Supplementary Table 1 and Fig. 2B). We also observed a significant main effect of parity and trends for an effect of stress and interaction. Post hoc analyses revealed that these effects were primarily driven by the P + S group, which exhibited significantly lower preference for a 1% sucrose solution compared to primiparous controls (Fig. 2B). There was no significant difference between C and S females in sucrose preference. Notably, there were no significant differences in total liquid consumption between groups (Supplementary Fig. 2C), ruling out differences due to overall consummatory behavior. We then assessed early postpartum anxiety-like behavior in the elevated plus maze. We found no significant interaction between stress and parity on percent time spent in open arms, and no main effects of either parity or stress (Fig. 2C). Additionally, there were no significant interactions or main effects of stress or parity on the number of entries into open arms, distance traveled, or average velocity (Supplementary Figs. 3A–C). Home cage maternal care observations during the light and dark cycles across the early postpartum period revealed that P + S females exhibited on average significantly fewer bouts of nursing (Fig. 2D) and licking/grooming (Fig. 2E), but no differences in consummatory or self-grooming behaviors (Supplementary Figs. 4A–B) compared to P counterparts. We also evaluated stress-coping strategy in a one-day, 15-min forced swim test in the mid-postpartum. We found a significant main effect of stress on both nulliparous and primiparous females in latency to immobility in the forced swim test, such that both S and P + S females had shorter latencies to immobility compared to respective nulliparous and primiparous controls (Fig. 2F). There was no main effect of parity nor interaction. Non-stressed primiparous dams showed no significant difference in latency to immobility compared to nulliparous controls. Taken together, these data replicate previously published findings (Brummelte and Galea, 2010; Leuner et al., 2014; O'Mahony et al., 2006; Pardon et al., 2000; Perani and Slattery, 2014) and demonstrate that our CMUS protocol was effective in altering postpartum behaviors.

### 3.2 Gestational stress reduces mitochondrial respiration in the prefrontal cortex

**Fig. 3:**
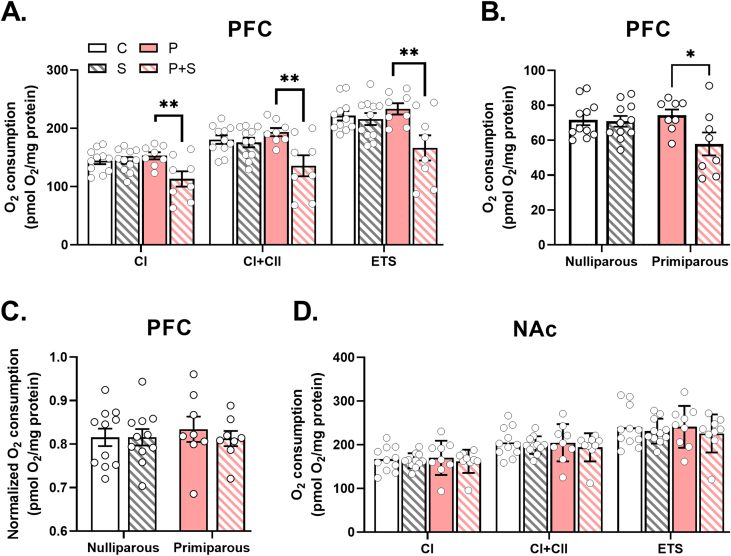
**Gestational stress decreases mitochondrial respiration in the prefrontal cortex but not nucleus accumbens**. **(A)** In prefrontal cortex (PFC), there was a significant interaction and main effect of stress, with no main effect of parity, on complex I (CI) (interaction: p = 0.01, stress: p = 0.02, parity: p = 0.15), complex I + II coupled (CI + CII) (interaction: p = 0.02, stress: p = 0.005, parity: p = 0.2), and maximal (ETS) (interaction: p = 0.02, stress: p = 0.007, parity: p = 0.14) respiration, where post hoc analyses demonstrated significantly decreased respiration in all measures in P + S dams compared to P dams ((CI) P vs. P + S: p = 0.004, (CI + CII) P vs. P + S: p = 0.002, (ETS) P vs. P + S**:** p = 0.003). n = 8–11/group. **(B)** In PFC, there was a trend for an interaction of stress and parity on complex II respiration (p = 0.06) and a significant main effect of stress (p = 0.04), but no main effect of parity (p = 0.21), although post hoc analyses revealed lower respiration in P + S compared to P dams (p = 0.02). n = 8–11/group. **(C)** There were no significant differences observed between groups in PFC mitochondrial content, as measured by CI + II/ETS-linked capacity (interaction: p = 0.62, stress: p = 0.63, parity: p = 0.72). n = 8–11/group. **(D)** In nucleus accumbens (NAc), there were no significant interactions or main effects of stress or parity on CI, CI + CII, or ETS respiration (see Supplementary Table 1 for statistical details). n = 9–11/group. C = nulliparous controls, S = nulliparous stressed, P = primiparous controls, and P + S = primiparous stressed. All data are represented as mean ± SEM. *p ≤ 0.05, **p ≤ 0.01, ***p ≤ 0.001, ****p ≤ 0.0001.

**Supplementary Fig. 5:**
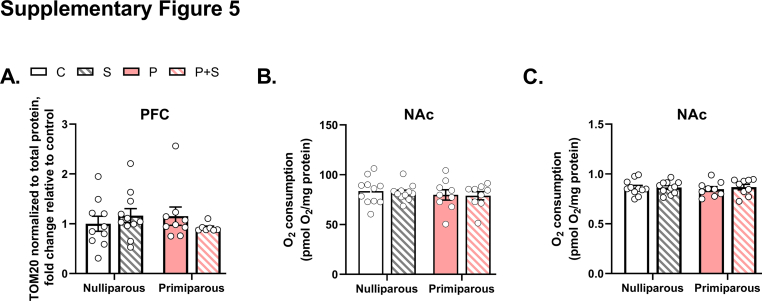
**No effects of stress or parity on mitochondrial content in PFC or NAc**. (A) There were no significant differences between groups in expression of the mitochondrial outer membrane protein, TOM20 in the prefrontal cortex. n = 7–11/group. **(B)** There were no significant interactions or main effects of stress or parity on complex II respiration in nucleus accumbens (NAc). n = 9–11/group **(C)** There were no significant differences in CI + II/ETS-linked capacity in the NAc between groups. n = 9–11/group. See Supplementary Table 2 for statistical details. C = nulliparous controls, S = nulliparous stressed, P = primiparous controls, and P + S = primiparous stressed. All data are represented as mean ± SEM.

We next evaluated the effects of stress and parity on mitochondrial function in the postpartum prefrontal cortex (PFC) and nucleus accumbens (NAc), two brain regions implicated in PPD (Pawluski et al., 2017; Ray et al., 2016). Using high-resolution respirometry, we found that gestational stress induced significant reductions in PFC mitochondrial respiration. Specifically, we observed significant main effects of stress and an interaction between stress and parity to decrease coupled respiration through complex I (CI) and complex II (CI + CII; Fig. 3A) in P + S females compared to P counterparts. When respiration was uncoupled, gestational stress significantly reduced the maximal electron transport system capacity (ETS; Fig. 3A). Upon complex I inhibition by rotenone, there was a significant main effect of stress and a trend for a parity by stress interaction to decrease maximal respiration due to complex II activity (ETS + CII) in P + S females, suggesting that respiratory differences observed in P + S females are driven by both complex I and II-dependent deficits in respiration (Fig. 3B). We analyzed CI + II/ETS-linked capacity, an indication of mitochondrial content (Burtscher et al., 2015), and found no effects of parity or stress on mitochondrial content (Fig. 3C), suggesting that the observed respiratory chain deficits were not due to differences in mitochondrial number. Similarly, analysis of mitochondrial outer membrane protein TOM20, a commonly used mitochondrial marker (Zhang et al., 2013), further revealed no differences between groups (Supplementary Fig. 5A). We did not observe any significant main effects of parity or stress on mitochondrial respiration in the NAc across any of our measures (Fig. 3D and Supplementary Fig. 5B), or on mitochondrial content (Supplementary Fig. 5C). suggesting that the effects of gestational stress on mitochondrial function are not global.

### 3.3 Gestational stress reduces mitochondrial complex I protein expression, with no effect on ROS

**Fig. 4:**
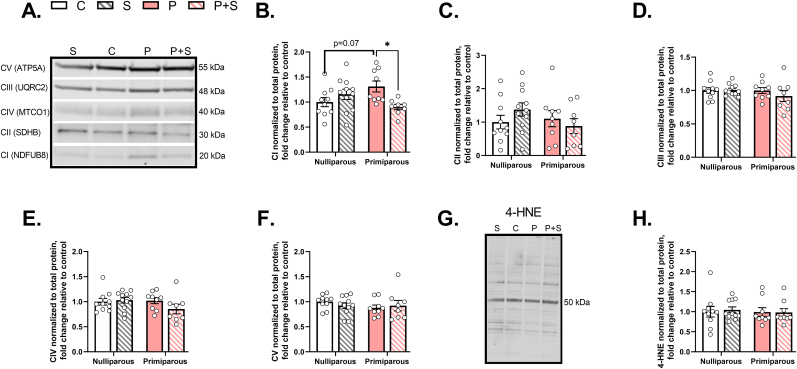
**Gestational stress decreases mitochondrial complex I protein but has no effect on other complexes or 4-HNE protein expression in prefrontal cortex. (A)** Representative Western blots of complex protein. **(B)** In prefrontal cortex, we found a significant interaction between stress and parity on complex I protein expression (p = 0.006), with no significant main effects of parity or stress, such that P dams exhibited a trend for increased protein expression compared to C females (C vs. P: p = 0.07), and P + S dams had significantly reduced protein expression compared to P dams (P vs. P + S: p = 0.01). No significant differences were observed between groups in protein expression of **(C)** complex II, **(D)** complex III, **(E)** complex IV, or **(F)** complex V. Similarly, **(G)** the representative 4-HNE western blot image demonstrates **(H)** there were no significant effects of stress, parity, or interaction on 4-HNE (see Supplementary Table 1 for statistical details). n = 8–11/group. C = nulliparous controls, S = nulliparous stressed, P = primiparous controls, and P + S = primiparous stressed, 4-HNE = 4-hydroxynonenal. All data are represented as mean ± SEM. *p ≤ 0.05, **p ≤ 0.01, ***p ≤ 0.001, ****p ≤ 0.0001.

Given the reduction in mitochondrial respiration in the PFC, we sought to determine whether gestational stress decreased mitochondrial complex protein levels. Immunoblots of PFC homogenates (Fig. 4A) revealed a significant interaction between stress and parity on complex I protein expression (Fig. 4B). We did not observe any significant main effects of parity, stress, or interaction on complex II, III, IV, or V protein levels (Fig. 4C–F), suggesting that the effect of parity and stress on mitochondrial complex protein may be specific for complex I. As stress has been shown to increase reactive oxygen species (ROS) and complex I is both a major site of ROS production and target for detrimental ROS actions (Musatov and Robinson, 2012; Simeone et al., 2014), we also examined the PFC for evidence of enhanced levels of 4-hydroxynonenal (4-HNE), a primary ROS-induced lipid peroxidation product. We did not observe any significant main effects of stress, parity, or interaction on 4-HNE levels (Fig. 4G–H), indicating that the observed decrease in mitochondrial respiration is not due to ROS-mediated lipid peroxidation, though this does not rule out possible effects of stress, parity, or interaction on other specific forms of ROS such as super oxides or hydrogen peroxides.

### 3.4 Gestational stress increases pro-inflammatory cytokine levels in the PFC and plasma that correlate with PFC mitochondrial respiration

**Fig. 5:**
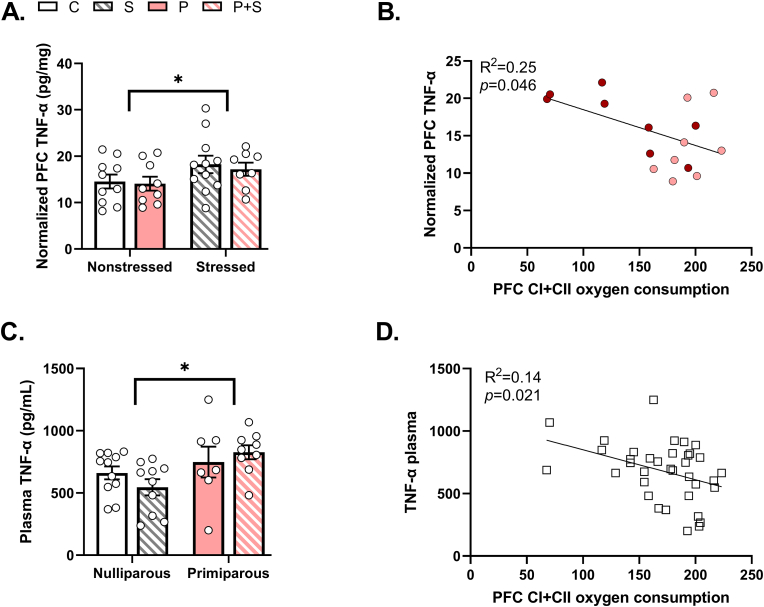
**Gestational stress decreases tumor necrosis factor alpha (TNF-α) levels in PFC and plasma. (A)** We found a significant main effect of stress on TNF-α in the prefrontal cortex (PFC), (p = 0.05), but no significant main effect of parity (p = 0.65), nor interaction (p = 0.86). Post hoc analyses demonstrated no significant differences between P and P + S dams. Note that order of groups on graph are different from other figures as to highlight the significant main effect of stress. n = 8–11/group. **(B)** In P (pink dots) and P + S (red dots) dams, CI + CII coupled respiration in the PFC was predictive of TNF-α in the PFC, where higher levels of the cytokine correlated with lower oxygen consumption. (*Simple Linear Regression*, n = 16). **(C)** In plasma, there was a significant main effect of parity on TNF-α (p = 0.02), while there was no significant interaction (p = 0.19) or main effect of stress (p = 0.8). However, post hoc analyses revealed no significant differences between groups. n = 7–11/group. **(D)** We observed that CI + CII coupled respiration in the PFC was predictive also of TNF-α levels in plasma, with inclusion of all groups, such that higher levels of plasma TNF-α were associated with lower levels of oxygen consumption. (*Simple Linear Regression*, n = 36). C = nulliparous controls, S = nulliparous stressed, P = primiparous controls, and P + S = primiparous stressed. All data are represented as mean ± SEM. *p ≤ 0.05, **p ≤ 0.01, ***p ≤ 0.001, ****p ≤ 0.0001. (For interpretation of the references to colour in this figure legend, the reader is referred to the Web version of this article.)

**Supplementary Fig. 6:**
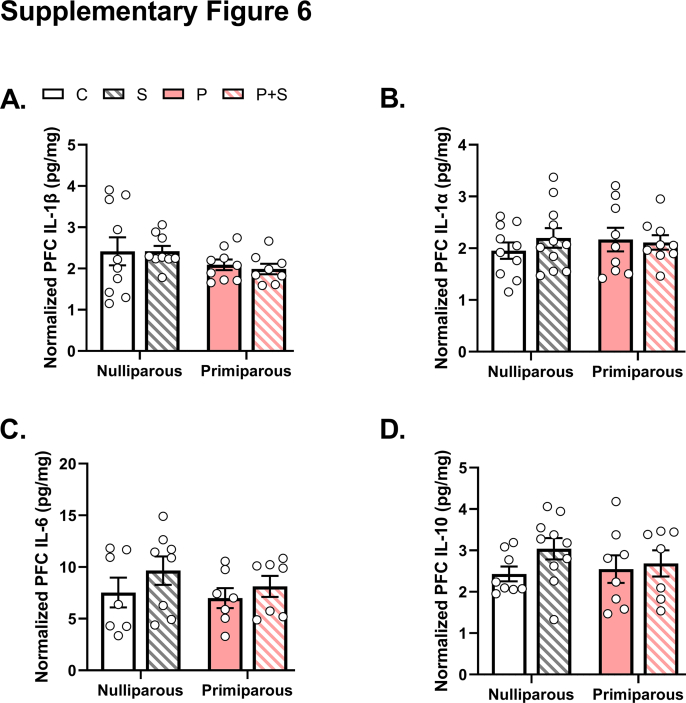
**There were no effects of parity or gestational stress on other measured PFC cytokine levels**. There were no significant main interactions, or effects of stress or parity on levels of **(A)** IL-1β, **(B)** IL-1α, **(C)** IL-6, **(D)** or IL-10 in the prefrontal cortex. n = 7–11/group. See Supplementary Table 2 for statistical details. C = nulliparous controls, S = nulliparous stressed, P = primiparous controls, and P + S = primiparous stressed. All data are represented as mean ± SEM.

**Supplementary Fig. 7:**
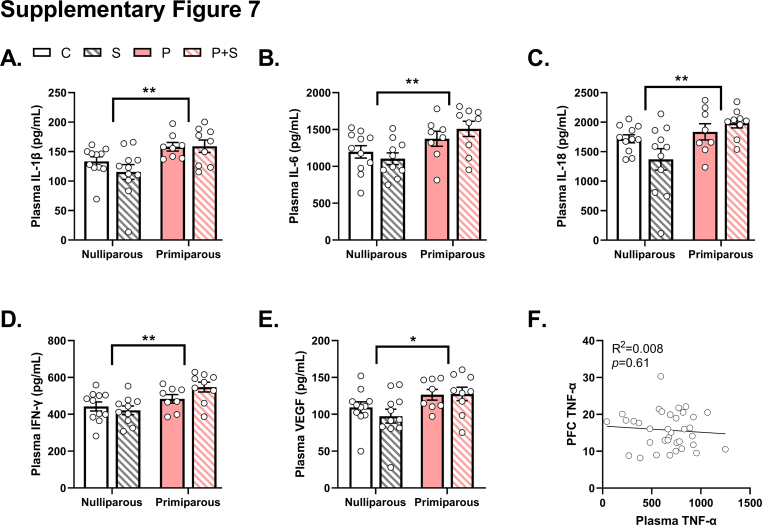
**Significant effect of parity on additional plasma cytokine levels**. (A) IL-1β levels exhibited a significant main effect of parity (p = 0.002), but post hoc analyses revealed no significant differences between groups. n = 8–11/group. **(B)** Analysis of IL-6 revealed a significant main effect of parity (p = 0.003). n = 8–11/group. **(C)** There was a trend for an interaction between parity and stress (p = 0.06) and a significant main effect of parity (p = 0.008), but no main effect of stress (p = 0.44) in IL-18 levels. Post hoc analyses revealed no significant differences between groups. n = 8–11/group. **(D)** Similarly, there was a trend for an interaction (p = 0.096) and a significant main effect of parity (p = 0.002) on IFN-γ levels. n = 8–11/group. **(E)** There was a significant main effect of parity (p = 0.01), but no effect of stress or interaction on VEGF levels. Post hoc analyses revealed no significant differences between groups. n = 8–11/group. See Supplementary Table 2 for statistical details. **(F)** TNF-α in prefrontal cortex does not correlate with TNF-α in plasma. (*Simple linear regression*, n = 37). C = nulliparous controls, S = nulliparous stressed, P = primiparous controls, and P + S = primiparous stressed. All data are represented as mean ± SEM. *p ≤ 0.05, **p ≤ 0.01, ***p ≤ 0.001, ****p ≤ 0.0001.

PPD has been linked with inflammation (Anderson and Maes, 2013; Dye et al., 2022) and mitochondrial dysfunction can result in the release of immunogenic compounds that activate inflammasomes to promote pro-inflammatory cytokine release (West and Shadel, 2017). Therefore, we examined cytokine levels in the plasma and PFC homogenates using BioPlex assays. In PFC, we found a significant main effect of stress on Tumor Necrosis Factor-alpha (TNF-α) (Fig. 5A). We did not observe any significant effects on levels of Interleukin (IL) 1-β, 1-α, 6, or 10 (Supplementary Figs. 6A–D). As these measures were taken from the same animals where mitochondrial respiration was measured, we performed linear regressions between mitochondrial respiration and TNF-α levels. We found a significant negative correlation between PFC TNF-α levels and coupled CI + CII respiration in primiparous rats, such that higher levels of TNF-α were associated with lower mitochondrial coupled respiration (Fig. 5B). In contrast, we observed a somewhat different cytokine profile in the plasma. In plasma, we found significant main effects of parity on levels of TNF-α (Fig. 5C), as well as levels of IL-1β, IL-6, IL-18, IFN-γ, and VEGF (Supplementary Figs. 7A–E). Given the increased TNF-α in both brain and periphery, we performed correlations to determine if plasma TNF-α levels might be associated with central TNF-α levels and/or PFC mitochondrial respiration. Surprisingly, we did not observe a significant correlation between plasma and PFC TNF-α levels (Supplementary Fig. 7F). However, we did observe a significant negative correlation between plasma TNF-α and PFC mitochondrial respiration (Fig. 5D), highlighting an exciting possible relationship between peripheral TNF-α levels and central mitochondrial function.

## 4 Discussion

Here we show that chronic gestational stress is associated with altered postpartum behaviors, decreased postpartum mitochondrial respiration in the prefrontal cortex (PFC) and increased inflammation at the central and systemic levels. In contrast, mitochondrial respiration in the nucleus accumbens (NAc) was unaffected by stress exposure or pregnancy. Our inclusion of both nulliparous and primiparous stress and non-stressed groups allowed us to dissect the individual effects of stress and pregnancy as well as their interaction on behavior, postpartum brain mitochondrial function, and inflammation. While mitochondrial involvement has been shown in other behaviors, including those related to social dominance (Hollis et al., 2015; van der Kooij et al., 2018), sociability (Morató et al., 2022), stress (Hara et al., 2014; Weger and Sandi, 2018), anxiety (Filiou et al., 2011; Filiou and Sandi, 2019), and depression (Allen et al., 2021; Gebara et al., 2021), our study is the first to characterize brain mitochondrial functional changes both in the postpartum period and following exposure to chronic gestational stress. Overall, these data highlight the potential for mitochondria to act as a mediator for the pathological effects of stress on behavior and inflammation in the postpartum period.

First, using a late-gestational, 10-day chronic mild unpredictable stress paradigm, we induced maternal care deficits, anhedonic, and passive stress-coping behaviors in female rats in the early to mid-postpartum period. Interestingly, we observed a main effect of parity in the sucrose preference – where only P + S females exhibited a decreased sucrose preference. While many studies using similar stress protocols have induced anhedonia in nulliparous animals (Franceschelli et al., 2014), in our hands CMUS exposure alone did not significantly affect sucrose preference. The most plausible explanation for this difference is the duration of our stress exposure, as a study by Baker et al. demonstrated limited effects of 3 weeks of chronic mild stress on nulliparous female sucrose preference (Baker et al., 2006), and most CMUS studies range from 3 weeks to 3 months in duration, employing multiple stress exposures in a single day (Franceschelli et al., 2014). Our protocol duration was necessarily limited to 10 days to restrict exposure to the late gestational period, with a single stress exposure per day. We did not observe any effects of parity or stress on anxiety-like behavior in the elevated plus maze, similar to some groups (Leuner et al., 2014), but in contrast to others (Darnaudéry et al., 2004; Hillerer et al., 2011). A likely explanation for this discrepancy is a difference in test lighting conditions, as we measure in low light to assess trait rather than state anxiety and studies suggest the need for high lighting levels to evoke anxiety in nulliparous control females (Lonstein, 2007). Additionally, anxiety phenotypes can vary according to test and timing. Thus, while we did not observe effects of gestational stress in the elevated plus maze the early postpartum, it is possible that other tests such as the open field or light-dark test may capture effects. Alternatively, previous work found that anxiety is dampened in the early postpartum (Lonstein, 2007). Thus, anxiety phenotypes may emerge in later postpartum time points. It is also important to note that while symptoms of anxiety are often comorbid with those of depression in human populations, they certainly do not always occur together (Austin et al., 2010; Ross et al., 2003). Importantly, both nulliparous and primiparous stressed females exhibited significantly decreased latencies to immobility in the forced swim test, in line with other reports using rodent stress models (Mir et al., 2022), suggesting that our protocol was stressful. The finding that our stress paradigm elicited specific effects (*e.g.*, reduced weight gain and sucrose preference) in primiparous, but not nulliparous, females suggests that pregnancy may increase susceptibility to developing maladaptive behaviors in this brief context of chronic mild stress.

The major finding of our study was the observation of specific differences in postpartum PFC mitochondrial function following gestational stress exposure. Gestationally stressed (P + S) but not primiparous non-stressed (P) or nulliparous stressed (S) dams exhibited a significant decrease in postpartum mitochondrial respiration in the PFC. While we measured respiration in tissue homogenates, our respiratory rates are strong and comparable to studies performed in isolated mitochondria (Kudin et al., 2004; Rekuviene et al., 2017). This decrease was not due to decreased mitochondrial number as mitochondrial content was similar between groups, as evidenced by a lack of difference between groups in PFC CI + II/ETS-linked capacity and TOM20 protein levels. Instead, we observed a significant decrease in complex I protein levels in the P + S group compared to P females, suggesting that gestational stress induces a depletion in respiratory complex I per mitochondria. The mechanism of depletion remains unclear, though ROS-dependent effects, including posttranslational modifications, have been previously shown to inhibit complex I mitochondrial respiration (Simeone et al., 2014). Notably, we did not observe any alterations in ROS-induced lipid peroxidation products between any of our groups. This may be due to the analysis of different ROS products (4HNE vs TBARS, superoxides, or hydrogen peroxide production), or the specificity for cellular compartments. As we analyzed the total homogenate, it is possible that mitochondrial-specific effects were diluted. Interestingly, P females tended to exhibit increased postpartum complex I protein levels compared to nulliparous controls, while P + S females notably did not. The increased protein level is intriguing as P females did not exhibit any difference in respiratory levels from nulliparous controls, highlighting the potential for a compensatory increase in protein expression to meet potentially increased metabolic demand in the postpartum period. While such studies in pregnant and postpartum women are limited, there is some evidence of pregnancy-driven physiological regulation of mitochondrial function, with pregnancy upregulating membrane potential to compensate for increased ROS production (Feldthusen et al., 2014). Taken together, our results suggest decreased PFC postpartum mitochondrial respiration in P + S females due to a lack of enrichment in complex I respiratory complex per mitochondria, compared to their P counterparts.

Interestingly, CMUS had no effect on mitochondrial respiration within the NAc of dams, indicating that postpartum brain mitochondrial respiration is not globally reduced by gestational stress. The NAc is largely involved in reward and motivation and plays a major role in maternal care during the postpartum, including pup-motivated behaviors (Champagne, 2004). We previously reported a link between anxiety-like behavior on the elevated plus maze and NAc mitochondrial function (Hollis et al., 2015). As we did not observe significant anxiety-like behaviors on the elevated plus maze, our lack of difference in NAc mitochondrial respiration further supports NAc mitochondrial respiration as a significant link to anxiety-like behavior on the elevated plus maze. Animal models of PPD typically demonstrate alterations in maternal care and have been linked to disruptions in NAc regulation (Champagne and Meaney, 2006; Pardon et al., 2000; Post and Leuner, 2019; Smith et al., 2004). Furthermore, in a study by Haim et al., chronic restraint stress between gestational days 7–20 induced changes in NAc structural plasticity during both the early/mid postpartum period (Haim et al., 2014). However, we did not observe any effects of parity or gestational stress on mitochondrial function within the NAc. It is possible that different types of stress can elicit varying phenotypes (Du Preez et al., 2021). For example, Haim and colleagues used homotypic chronic restraint stress while we employed a heterotypic stress protocol, using unpredictable stressors at different times of the day. It is also possible that a longer duration of stress would induce alterations that we do not observe with a 10-day protocol, or that differences would be observed in an earlier or later postpartum time point. Finally, there could be population-specific changes that we are unable to detect as we did not sub-divide the NAc into core and shell regions. Future studies that examine the effects of different types of stressors, additional time points, or specific cellular populations will allow us to further understand the specificity of effects of gestational stress on brain mitochondrial function.

The lack of effect in NAc also highlights the PFC as a region particularly susceptible to the remodeling effects of gestational CMUS. The PFC has consistently been implicated in preclinical studies in rodent models of stress and depression (Duman, 2014) and associated with decreased volume, neuronal atrophy, and altered connectivity in stress-related pathologies (Drevets et al., 2008). Furthermore, our findings corroborate other rodent studies reporting brain mitochondrial susceptibility to chronic stress. For instance, a study by Weger and colleagues observed chronic stress-induced mitochondrial gene signature alterations accompanied by reduced respiration in the PFC but not the NAc (Weger and Sandi, 2018). While these studies were primarily in males, used a longer duration of stress, and did not explore the contributions of pregnancy, they similarly support higher susceptibility of the PFC compared to the NAc to chronic stress, which could account for the decreased respiration we observe under our CMUS paradigm. Importantly, the PFC is one of several known brain regions affected by pregnancy, exhibiting volumetric (Hoekzema et al., 2017) and morphological (Leuner and Gould, 2010) changes suggestive of a role in maternal behaviors (Hillerer et al., 2014), and involvement in clinical (Moses-Kolko et al., 2010) and preclinical (Leuner et al., 2014) models of PPD. Moreover, other studies using gestational stress models have reported enduring reductions in PFC dendritic spine density and morphology that was associated with PPD-relevant behaviors such as maternal care and passive coping (Haim et al., 2016; Leuner et al., 2014). Mitochondria localize to synapses to provide crucial energy and buffering capacity during synaptic activity (Rangaraju et al., 2019), and artificial depletion of mitochondria results in decrease in dendritic spines and synapse density (Li et al., 2004). Thus, our observed reductions in mitochondrial respiration may underlie the stress-induced structural remodeling reported by others, though further studies are necessary to concretely link these two processes.

Immune system alterations have been noted in both pregnancy and the postpartum period. While normal pregnancy is characterized by an anti-inflammatory state with pro-inflammatory shifts depending on the stage (Bränn et al., 2019), stress during pregnancy can alter the immunological profile of mothers, promoting a more pro-inflammatory state that is associated with PPD (Anderson and Maes, 2013; Christian, 2015). Stress may also damage mitochondria, initiating the release of ROS or mitochondrial DNA, which further promotes a pro-inflammatory response via inflammasome activation and cytokine production (Riley and Tait, 2020; Zhou et al., 2011). We examined cytokine levels in both the PFC and plasma to determine whether our gestational stress protocol induced inflammation in association with mitochondrial dysfunction. In the PFC, stress significantly increased levels of TNF-α and these levels negatively correlated with PFC mitochondrial respiration. In the periphery, parity increased levels of TNF-α, IL6, IL-1β, IL-18, IFN-γ, and VEGF. Additionally, plasma TNF-α, but not other measured cytokines similarly correlated with PFC mitochondrial respiration. Despite the increased levels of TNF-α in both plasma and PFC and their significant correlations with PFC mitochondrial respiration, there was no significant correlation between neural and systemic TNF-α. This finding replicates previous studies reporting a lack of correlation between central and peripheral immune activation (Boufidou et al., 2009; Miller et al., 2019) and further highlights the unique and complex nature of postpartum inflammation. Indeed, this complexity is further underlined by the conflicting reports on gestational stress-induced inflammation at both the central and peripheral levels in the rodent literature (Dye et al., 2022), possibly due to differences in the timing of measurement and stress protocols. Measurements taken at GD21 identified increased levels of IL-1β and Interferon gamma (IFN-γ) in the PFC of gestationally stressed females, but no changes in plasma cytokine levels from non-stressed pregnant counterparts (Lenz et al., 2019). A separate study found increased IL-6 and decreased IL-1β in the PFC in gestationally stressed postpartum rats the day after parturition but did not measure in the periphery (Posillico and Schwarz, 2016). One month postpartum, O'Mahoney and colleagues observed increased TNF-α, IL-1β, and IL-10 levels in the plasma of gestationally stressed rats but did not observe any differences in the PFC (O'Mahony et al., 2006). These studies together with our findings here suggest a potential timeline of stress-induced neuroimmune changes during gestation that possibly develop into systemic inflammation in the postpartum. Longitudinal sampling will be necessary to fully establish the sequence of events surrounding stress-evoked increases in inflammation. Studies in patients, while limited, are slightly more consistent, with PPD women exhibiting enhanced TNF-α both systemically (Boufidou et al., 2009) and centrally (in cerebrospinal fluid) and these levels correlating with depressive symptoms (Boufidou et al., 2009). Moreover, levels of serum IL-6R were increased in postpartum women with increased depression scores relative to prenatal time points (Maes et al., 2000) and plasma IL-18 was increased in women 8 weeks postpartum (Bränn et al., 2020). Our cytokine data, coupled with these studies, lend further support to TNF-α as a cytokine that warrants further examination.

Importantly, our findings reveal a relationship between PFC mitochondrial function, inflammation, and PPD-relevant behaviors. We identified significant negative correlations between PFC mitochondrial respiration and TNF-α levels in the PFC and plasma. While the nature of our study does not allow us to draw conclusions on causality or directionality, it opens the door to several possibilities. One is that gestational stress has parallel effects on mitochondria, behavior, and inflammation. Another is that gestational stress induces inflammation that induces mitochondrial and behavioral dysfunction. The more tantalizing possibility, however, is that gestational stress alters postpartum behaviors and inflammation via effects on brain mitochondrial function. We have previously shown that mitochondrial function in specific brain regions can drive behavioral responses (Hollis et al., 2015, 2018; van der Kooij et al., 2018), so it is plausible that mitochondria act as mediators to link stress with behavioral consequences, however future studies are needed to tease apart this relationship. Moreover, mitochondria have been recently shown to activate inflammasomes and trigger the release of pro-inflammatory cytokines (West, 2017). The significant relationship between plasma cytokine levels and PFC mitochondrial respiration is particularly exciting as it suggests that plasma TNF-α may act as a biomarker for central mitochondrial function, and possibly, PPD. Further studies will be necessary to fully characterize the biomarker potential of TNF-α for PFC mitochondrial function and PPD. As cytokines can also act to disrupt mitochondrial function (Hollis et al., 2022) future studies that manipulate mitochondrial function will be needed to determine the precise role of PFC mitochondrial respiration in postpartum behaviors relevant for PPD and inflammation.

It is important to note that our mitochondrial measures were performed at a single mid-postpartum time point. Thus, we cannot rule out potential effects of dam-offspring interactions that may elicit neuronal changes following gestational stress (Baker et al., 2008; Champagne and Meaney, 2006; Leuner et al., 2014; Smith et al., 2004), nor potential interactions with hormones stimulating lactation, particularly as we observed differences in maternal nursing and grooming behaviors. Moreover, our study does not identify the precise time point at which mitochondrial respiration was affected. We do not know whether mitochondrial alterations occurred following parturition or developed across gestation with successive stress exposures. Nor do we know how long these changes persist in the postpartum period. Follow-up experiments that identify the timing, duration, and specificity of mitochondrial alterations will be necessary to clarify these points. Despite these limitations, our study represents an important first step in establishing the effects of gestational stress and pregnancy to alter postpartum brain mitochondrial function.

Since mitochondria are involved in many biological processes, the mechanisms contributing to our observed results could be multi-faceted. Importantly, in the current study, mitochondrial dysfunction was only observed when both stress and pregnancy were involved, pointing to interactions between the HPA and HPG axes. Pregnancy and parturition are marked by dramatic fluctuations in reproductive hormones, such as estrogen that decreases drastically in the postpartum (Brummelte and Galea, 2016). 17*β****-***Estradiol (E_2_) has been shown to benefit mitochondrial function by reducing oxidative damage and promoting energy production via oxidative phosphorylation (OXPHOS) (Brinton, 2008), and E_2_ affects stress responses of the hypothalamic-pituitary-adrenal (HPA) axis (Handa et al., 2012). Estrogen receptors can localize to mitochondria, where their actions can affect respiratory activity, mitochondrial enzymatic activity, and reduce lipid peroxidation (Irwin et al., 2012; Yang et al., 2004, 2009). It is possible that the fluctuation of estrogen levels during and shortly following pregnancy have downstream effects on mitochondrial function, promoting a dysfunctional phenotype. Similarly, corticosterone may also have a mechanistic role, considering that fluctuations in this hormone also occur during pregnancy (Brummelte and Galea, 2016) and glucocorticoid receptors can localize to mitochondria and regulate their function (Du et al., 2009). While these hypotheses will provide a crucial stepping-off point to future research, there are certainly other mechanisms to consider and the possibility of a combination of these processes should not be ruled out.

## 5 Conclusion

Despite the multitude of connections between mitochondrial function, stress and pregnancy, to our knowledge, this is the first study to directly examine brain mitochondrial function in pregnancy or following gestational stress. Overall, our results suggest that mitochondria could act as an upstream regulator of stress-induced processes which affect behavioral output. However, future studies to identify potential downstream targets, or whether dysregulation is occurring upstream that has direct consequences on mitochondrial function, are required to clarify the mechanisms involved. Finally, experiments in which mitochondrial function is either enhanced or reduced (*e.g.,* with treatments of an antioxidant or an electron transport system complex inhibitor, respectively) would provide necessary information on the direct role of these organelles on the manifestation of postpartum depressive-like behaviors. With these aims in mind, mitochondria could be a potential therapeutic target in the development of more effective and cost-efficient treatment for PPD patients, specifically for pregnant individuals who are more vulnerable to the combined effects of stress and pregnancy.

## Funding support

This work was supported by the Department of Veteran Affairs through VISN7 Research Development Award; as well as the 10.13039/100000002NIH with a COBRE Target Faculty award (P20GM109091) to FH and NIH RO1DK132948 grant to R.C.W.; USC institutional funds via a SPARC grant to EGS and a 10.13039/100008899UofSC ASPIRE I award and institutional start-up funding to FH.

## CRediT authorship contribution statement

**Erin Gorman-Sandler:** Conceptualization, Investigation, Formal analysis, Data curation, Writing – original draft, Writing – review & editing. **Breanna Robertson:** Investigation, Formal analysis, Data curation. **Jesseca Crawford:** Investigation. **Gabrielle Wood:** Formal analysis, Data curation. **Archana Ramesh:** Formal analysis. **Olufunke O. Arishe:** Investigation. **R. Clinton Webb:** Writing – review & editing. **Fiona Hollis:** Conceptualization, Formal analysis, Data curation, Writing – original draft, Writing – review & editing, Funding acquisition.

## Declaration of competing interest

The authors have no competing interests or conflict to declare.

## Data Availability

Data will be made available on request.
